# Effect of different optimization parameters in single isocenter multiple brain metastases radiosurgery

**DOI:** 10.1007/s00066-024-02249-z

**Published:** 2024-07-08

**Authors:** Angelika Altergot, Carsten Ohlmann, Frank Nüsken, Jan Palm, Markus Hecht, Yvonne Dzierma

**Affiliations:** Department of Radiotherapy and Radiation Oncology, Saarland University Medical Center, Kirrberger Straße, Homburg/Saar, Germany

**Keywords:** Elements Multiple Brain Mets, Brain neoplasms, Flat vs FFF beams, Gradient index, Plan complexity

## Abstract

**Purpose:**

Automated treatment planning for multiple brain metastases differs from traditional planning approaches. It is therefore helpful to understand which parameters for optimization are available and how they affect the plan quality. This study aims to provide a reference for designing multi-metastases treatment plans and to define quality endpoints for benchmarking the technique from a scientific perspective.

**Methods:**

In all, 20 patients with a total of 183 lesions were retrospectively planned according to four optimization scenarios. Plan quality was evaluated using common plan quality parameters such as conformity index, gradient index and dose to normal tissue. Therefore, different scenarios with combinations of optimization parameters were evaluated, while taking into account dependence on the number of treated lesions as well as influence of different beams.

**Results:**

Different scenarios resulted in minor differences in plan quality. With increasing number of lesions, the number of monitor units increased, so did the dose to healthy tissue and the number of interlesional dose bridging in adjacent metastases. Highly modulated cases resulted in 4–10% higher V_10%_ compared to less complex cases, while monitor units did not increase. Changing the energy to a flattening filter free (FFF) beam resulted in lower local V_12Gy_ (whole brain-PTV) and even though the number of monitor units increased by 13–15%, on average 46% shorter treatment times were achieved.

**Conclusion:**

Although no clinically relevant differences in parameters where found, we identified some variation in the dose distributions of the different scenarios. Less complex scenarios generated visually more dose overlap; therefore, a more complex scenario may be preferred although differences in the quality metrics appear minor.

## Introduction

Increasing evidence suggests that whole brain radiotherapy (WBRT) leads to radiation-induced early neurocognitive impairment and more specifically that low-dose irradiation of the hippocampus can lead to a blockade of neurogenesis and hence to short-term memory impairment [1–5]. Therefore, even for multiple brain metastases, stereotactic radiosurgery (SRS) is increasingly preferred over WBRT, with the aim of achieving better protection of normal brain tissue and preserving neurocognition in the patient without compromising local control and overall survival [6–13].

As imaging techniques evolve and patient positioning becomes more precise, treating multiple metastases with a single isocenter has become a commonly used concept for SRS [14, 15] rather than treating each metastasis with an individual plan (and mostly on different treatment days). The use of dedicated planning systems or specialized optimizers within general treatment planning systems (TPS) for multi-metastases stereotactic treatment offers a possibility to jointly optimize the dose distribution and therefore avoid interlesional dose bridging, which allows for improved normal tissue protection. Furthermore, it shortens the duration of the treatment itself [16] and the effort for plan optimization compared to multi-isocentric SRS [17, 18].

In recent years, flattening filter free (FFF) beams have become common especially for SRS due to their higher dose rate and hence reduced beam-on time, translating to a reduced treatment time in general [19]. Further advantages are reduced scatter and out-of-field dose [20]. While FFF beams are ideally suited for single-lesion stereotactic treatments since these require such small field openings that the FFF beam can be considered virtually like a flat beam with increased dose rate, these improvements are hampered by the necessity of increasing modulation of the “un-flat” fields in case of extended target lesions. Therefore, it is not evident whether this advantage persists for mono-isocentric treatment of multiple metastases, which may be located at different distances from the central axis and therefore receive different beam intensities.

Automated treatment planning for multiple metastases with a single isocenter is available for several treatment planning systems [21], one of them Brainlab’s Multiple Brain Mets SRS (MBM) in Elements (Version 3.0, Brainlab, Munich, Germany) [18]. This system still offers different parameter settings for the number of applied arcs, modulation and target coverage, which requires iterative planning and optimization by the user, and which may result in plans of different quality and rather different treatment efficiency in terms of on-couch treatment time. The aim of this paper was therefore to systematically compare these different parameter settings, while at the same time evaluating the influence of flat vs. FFF beams.

This paper will focus on the question of how best to design these multi-metastases single-isocenter treatment plans, as well as from a scientific perspective to define the quality endpoints for benchmarking the technique.

## Materials and methods

### Patient selection

In our retrospective study, 20 patients with brain metastases were randomly selected and replanned using four different scenarios for comparison. The number of metastases ranged from 3 to 28 per patient with a lesion diameter of less than 20 mm, giving a total of 183 lesions. To delineate the target volumes, i.e., the gross tumor volume (GTV), the planning target volume (PTV) and the organs at risk (OAR), a contrast-enhanced T1 MP-RAGE sequence with an axial slice thickness of 1 mm was used. In this study, the healthy brain tissue and hippocampus were considered as organs at risk and were retrospectively evaluated as a measure for plan quality, but were not considered in the optimization process.

Different constellations were observed in our study. In a first step we evaluated the plan quality by single target volumes (TV), not considering how many were treated with a single isocenter. In the next step, we evaluated the data patient-wise and with a subgroup analysis of patients with few medium and many lesions (Table 1). The average volume of all GTVs was 0.20 ± 0.37 cm^3^ (range 0.01–2.99 cm^3^) and the average distance from the center of the lesions to the isocenter was 5.28 ± 1.57 cm (range 1.30–9.40 cm).

**Table 1:** Definition of the subgroups

	“all”	“few”	“medium”	“many”
Number of lesions	3–28	3–5	6–10	> 10
Number of patients	20	8	7	5

### Treatment planning system

For treatment planning, we used the dedicated treatment planning module Multiple Brain Mets in the treatment planning system (TPS) Elements. Our system is commissioned for the TrueBeam accelerator (Varian Medical Systems, Palo Alto, CA, USA) with Millennium multi leaf collimator (MLC, 5 mm leaf width) with an energy of 6 MV with and without flattening filter (FFF) and with a dose rate of 600 monitor units per minute (MU/min) and 1400 MU/min, respectively. We chose a dose of 20 Gy for prescription/coverage dose and a maximum point dose of 25 Gy according to our in-house standard (20 Gy to the encompassing 80% isodose with a point dose of 25 Gy) for single fraction stereotactic radiosurgery of metastases smaller than 20 mm in diameter and not in close proximity to organs at risk [7]. Based on a preconfigured prescription protocol and a setup protocol, this module automatically configures noncoplanar dynamic conformal arcs (DCA) for single isocenter irradiation of multiple brain metastases. The isocenter is placed in the center of mass of all metastases. In the prescription protocol, desired and tolerated coverage volume were set to 99%, meaning that 99% of the target volume must receive the prescribed dose. Tolerated dose deviation was set to 2%, meaning that a maximum dose or overdosage up to 102% of the prescribed dose is allowed. All plans were optimized with a specific parameter setting which allows the specified overdosage within the target volumes if this is required to achieve target coverage (‘SRS prescription (controlled inhomogeneity)’ on). Automatic margin generation was selected, which creates planning target volumes (PTVs) from delineated gross tumor volumes (GTVs) with a preconfigures margin. Margins were either set at 1 mm regardless of the GTV-to-isocenter distance [14, 22, 23] or increased stepwise in relation to the GTV-to-isocenter distance [24]. In the latter case, no margin was created for less than 10 mm GTV-to-isocenter distance, 1 mm for a distance between 10 mm and 50 mm and a 2 mm margin for a GTV-to-isocenter distance greater than 50 mm. The dose grid spatial resolution was set to 1 mm and Monte Carlo (MC) statistical uncertainty for final forward dose calculation was set to 1%. Healthy brain tissue was defined as volume of the brain excluding the target volumes (whole brain-PTV). The setup protocol allowed for five different couch angles.

For better comparability of the results, optimization was carried out in a fixed order. First, all parameter settings were selected for the specific scenario, then a first optimization run is performed with the pencil beam algorithm (this is required in this TPS before moving on to MC). A recalculation of the same plan with the MC algorithm is then carried out, after which a second optimization run is performed. At least these three steps are needed for optimization in MBM Elements. No further parameters were changed during these defined workflows.

Four scenarios with different settings were examined.

For scenario 1, no extra arcs were enabled, the DCA complexity was set to low and a 1 mm margin was chosen for all GTV-to-isocenter distances. The complexity, i.e., the degrees of freedom in the modulation and therefore the number of monitor units, can be limited using the DCA complexity toggle. The lowest setting of the DCA complexity toggle means greatest restriction on modulation, while the highest setting entails the least restriction on modulation and plan complexity. In our study, we considered two extreme settings—for scenario 3, the complexity was set to high, i.e., allowing highly complex plan creation.

Scenario 2 was equal to the first one; however, we allowed extra arcs to be added. In our study, these extra arcs were assigned to the three volumetrically largest lesions, since this version does not include hybrid optimization with additional volumetric intensity modulated arc therapy (VMAT) arcs.

Scenario 3 was also the same as the first (i.e., again no extra arcs), but now the DCA complexity was set to “high”.

In the final scenario 4, we aimed to observe the effect of margin expansion and therefore chose a prescription template with stepwise increasing margins of 0, 1 and 2 mm depending on the GTV to isocenter distance. DCA complexity was set to low and we allowed additional arcs, similar to scenario 2. All scenarios and their corresponding settings are summarized in Table 2 below.

**Table 2:** Definition of all four settings used in this study

	Extra arcs	DCA complexity	Margin (mm)	Replanned with FFF
Scenario 1	No	Low	1	No
Scenario 2	Yes	Low	1	Yes
Scenario 3	No	High	1	Yes
Scenario 4	Yes	Low	0, 1, 2	No

*DCA* dynamic conformal arcs, *FFF* flattening filter free

**Fig. 1:**
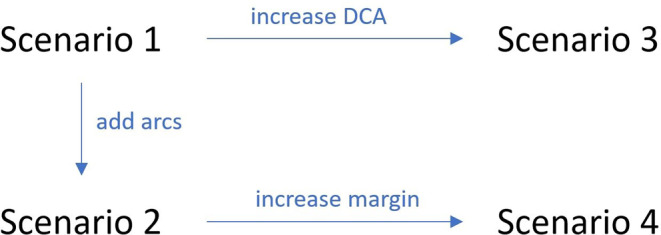
Diagram for visualization of all compared scenarios and the corresponding changes made within these scenarios. (*DCA* dynamic conformal arcs)

In our study we only modified one variable at a time. Scenario 1 and 2 compare the effect of adding more arcs, scenario 1 and 3 differed in allowed plan complexity, and scenario 2 and 4 contrast different margin recipes. Other pairwise comparisons would lead to more than one modification and have therefore not been included. A diagram is shown in Fig. 1.

All patients in all four scenarios were first optimized with a 6 MV flattened beam. In a second approach, we recalculated those plans for scenario 2 and 3 with a flattening filter free 6 MV beam. Pairwise comparison was then performed for both scenario 2 and 3.

### Plan evaluation and statistical analysis

Apart from global V_12Gy_ of the healthy brain tissue (minus PTVs), dose–volume histogram (DVH) statistics for the PTV such as D_99%_, as well as OAR were evaluated [25, 26]. The V_12Gy_ has been included since its volume has been correlated with the risk of developing radionecrosis [27–30]. It is generally presumed that the V_12Gy_ should be evaluated locally for each metastasis, as long as these are far enough apart so that no dose bridging occurs. In clinical practice, we would evaluate all local V_12Gy_ values individually to ascertain that our in-house objective V_12Gy_ < 10 cm^3^ is not exceeded. Since this was always the case in the plans created for the sake of this study, we opt to present here a global V_12Gy_ as a cumulative measure of plan quality for comparison of the V_12Gy_ doses to the brain outside the planned PTV, which will naturally be encompassed by the prescribed dose of 20 Gy.

The TPS provides a volume average conformity (CI) and gradient index (GI) as well as local CI and GI for each metastasis. For our analysis we used Paddick CI [31]. Indices are defined as follows:

1 $$\text{Paddick}\,\mathrm{CI}=\left(TV_{\mathrm{PIV}}\right)^{2}/(\mathrm{TV} \times \mathrm{PIV})$$

2 $$\mathrm{GI\ }=\mathrm{PIV}_{50}/\mathrm{PIV}_{100}$$

where TV is the volume of the PTV, PIV is the prescribed isodose volume, i.e., the volume of the surrounding 20 Gy isodose, TV_PIV_ is the volume of overlap between the target volume and the prescribed isodose volume. PIV_50_ defines the volume of the 50% prescribed isodose (in this case 10 Gy) and PIV_100_ is defined as the volume of the 100% prescribed isodose [32].

For statistical analyses, Origin Pro (Version 2022b, OriginLab Corporation, Northampton, MA, USA) was used. Average values were calculated for each parameter and scenario. These were compared pairwise via Wilcoxon signed rank test for paired data to show whether differences between the various settings were given. Wilcoxon singed rank test was chosen rather than the t‑test, since it is a nonparametric test not requiring a hypothesis regarding the distribution function of the data and since a normal distribution could not be presumed. First, plan evaluation was carried out considering all lesions of all patients together. Then, we checked the subgroups to identify effects arising from the number of lesions within a single-isocenter plan.

## Results

### All target volumes

In the following section, we analyze all target volumes nonregarding the number of metastases per patient to determine differences in the various scenarios for a flattened 6 MV beam without further dependence on the number of treated lesions per plan.

Whenever additional arcs were allowed, the TPS indeed made use of these additional degrees of freedom. Thus, for scenario 1 and 3, i.e., when no extra arcs were allowed, the overall minimum number of used arcs was 7. For scenario 2 and 4, i.e., with extra arcs, at least 11 and 12 arcs were used, respectively. The maximum was 14 arcs for all scenarios, which is also the largest possible number of arcs.

**Fig. 2:**
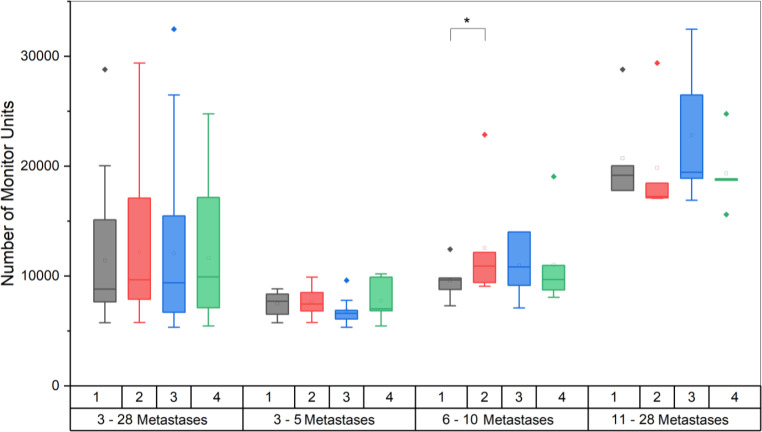
Boxplots showing the number of monitor units needed for the different scenarios for all plans and the subgroups separately. There was a significant difference between scenario 1 and 2 in subgroup B. (*p = 0.036)

Despite this fact, the number of monitor units (MU) is not significantly different between the scenarios. Mean values range between 11,434.0 ± 6067.6 MU and 12,183.9 ± 6250.7 MU, the minimum is between 5339.0 MU and 5757.0 MU and maximum values fall between 24,770.0 MU and 32,472.0 MU. Maximum values were all attained for the same patient (patient #17, 28 metastases) for all four scenarios. The left-hand side of Fig. 2 shows the number of monitor units per scenario for all metastases.

Increasing the margin as a function of the GTV-to-isocenter distance (scenario 4) significantly increases the mean cumulative volume of PTVs from 3.35 cm^3^ to 4.89 cm^3^ (*p* < 0.001).

Volume coverage was evaluated using the dose covering 99% of the PTV, i.e., D_99%_. The minimum D_99%_ within all scenarios was 20 Gy, which was input as an obligatory objective and was always satisfied in the optimization due to the planning technique.

**Fig. 3:**
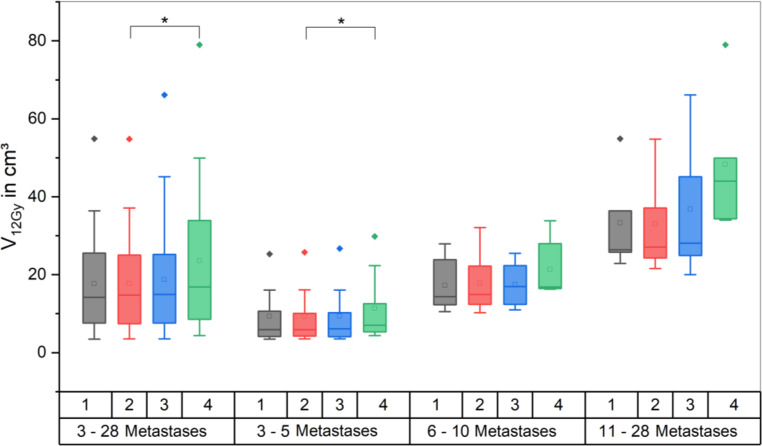
Boxplots showing the cumulative V_12Gy_ of the healthy brain minus planning target volume (PTV) for the different scenarios for all plans and the subgroups separately. (* significant at *p* < 0.05)

No significant differences in global V_12Gy_ were found in the comparison of scenario 1 vs. 2, and 1 vs. 3, respectively. Cumulative V_12Gy_ for scenario 4 with a mean value of 23.57 cm^3^, however, was significantly higher (*p* < 0.00013) when compared with scenario 2 with a mean V_12Gy_ of 17.78 cm^3^, which is plausible, considering that the target volumes are significantly larger. A visualization of this data is shown in Fig. 3.

For sparing of the hippocampus, there was no significant difference for the mean or maximum dose when comparing the scenarios 1 vs. 2 or 1 vs. 3. However, for scenarios 2 vs. 4 we saw a significantly different mean dose, where scenario 2 yielded lower doses to the hippocampus (2.13 ± 1.84 Gy) than scenario 4 (2.35 ± 1.64 Gy, *p* = 0.013). The different subgroups show a tendency to higher mean doses the larger the number of lesions is per plan. This is plausible as the overall dose in the brain, i.e., the low-dose bath, rises the more metastases are irradiated.

In scenario 4, the maximum dose was 4.57 ± 3.82 Gy which compared to 4.04 ± 3.98 Gy in scenario 2 was significantly higher (*p* = 0.004). This effect is once again caused by the larger target volumes in scenario 4.

**Fig. 4:**
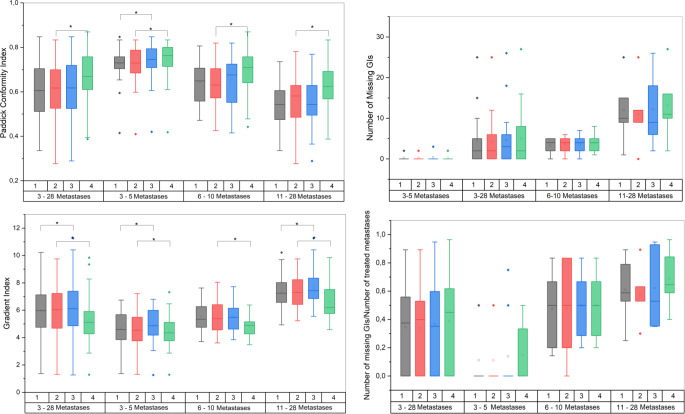
Boxplots showing the Paddick conformity index (CI), gradient index (GI), the number of missing GIs and the normalized number of missing GIs for the different scenarios for all plans and the subgroups separately. (* significant at *p* < 0.05)

Adding arcs or changing the DCA complexity did not result in better local Paddick CI. In contrast, margin increase showed a slight but statistically significant improvement from an average local CI of 0.61 for scenario 2 to 0.67 for scenario 4 (*p* < 0.0001) as shown in Fig. 4. Figure 4 also shows gradient indices which were similar for scenario 1 vs. scenario 2, whereas for comparison of scenario 1 and 3 a significant increase from an average local GI of 5.98 in scenario 1 to 6.15 in scenario 3 was found (*p* < 0.0005). Increasing the margin in scenario 4 improves the average local GI to 5.31 compared to 5.97 in scenario 2 (*p* < 0.0001). Global CI and GI values were slightly different for the scenarios as shown in Table 3. While the mean global GI was better in scenario 1 compared to scenario 3, a better CI was achieved in scenario 3. Again, coming from the larger target volumes, the best CI and GI values were reached for scenario 4.

**Table 3:** Mean volume average conformity index (CI) and global gradient index (GI) over all lesions within a plan for all subgroups and scenarios

		Scenario 1	Scenario 2	Scenario 3	Scenario 4
All	CI	0.720 ± 0.048 (11)^a^	0.722 ± 0.049 (11)^a^	0.736 ± 0.058 (11)^a^	0.759 ± 0.034 (10)^a^
	GI	4.571 ± 0.492 (7)^a^	4.508 ± 0.455 (8)^a^	4.681 ± 0.481 (7)^a^	4.277 ± 0.403 (7)^a^
Few	CI	0.741 ± 0.040 (6)^a^	0.745 ± 0.037 (6)^a^	0.767 ± 0.032 (6)^a^	0.767 ± 0.025 (6)^a^
	GI	4.660 ± 0.477 (6)^a^	4.600 ± 0.481 (6)^a^	4.780 ± 0.450 (6)^a^	4.353 ± 0.385 (6)^a^
Medium	CI	0.680 ± 0.037 (4)^a^	0.689 ± 0.040 (3)^a^	0.690 ± 0.063 (4)^a^	0.732 ± 0.035 (4)^a^
	GI	not calculable (0)^a^	4.42 ± 0 (1)^a^	not calculable (0)^a^	not calculable (0)^a^
Many	CI	0.752 ± 0 (1)^a^	0.703 ± 0.054 (2)^a^	0.711 ± 0.053 (2)^a^	0.794 ± 0 (1)^a^
	GI	4.04 ± 0 (1)^a^	4.05 ± 0 (1)^a^	4.09 ± 0 (1)^a^	3.82 ± 0 (1)^a^

^a^Number of calculable indices

Due to the leaf width, sufficient coverage is more easily achievable for larger target volumes, resulting in a steeper gradient (i.e., GI closer to 1) in scenario 4 compared to the smaller margin approaches.

The system is unable to generate a GI whenever the 50% isodoses (in this case 10 Gy) of two or more metastases overlap. We used this information to obtain a parameter for “dose bridges”, regions of isodose confluence between two metastases by counting the number of missing GIs.

Figure 4 shows that while this parameter is similar when looking at all metastases, there was a tendency for the subgroups to have more dose bridges the more metastases were planned with a single isocenter, which is plausible given the larger number of lesions and resulting closer spatial proximity (and also, possible statistical permutations). Therefore, we additionally considered a scaled value by dividing the number of dose bridges by the number of metastases within a single plan.

**Fig. 5:**
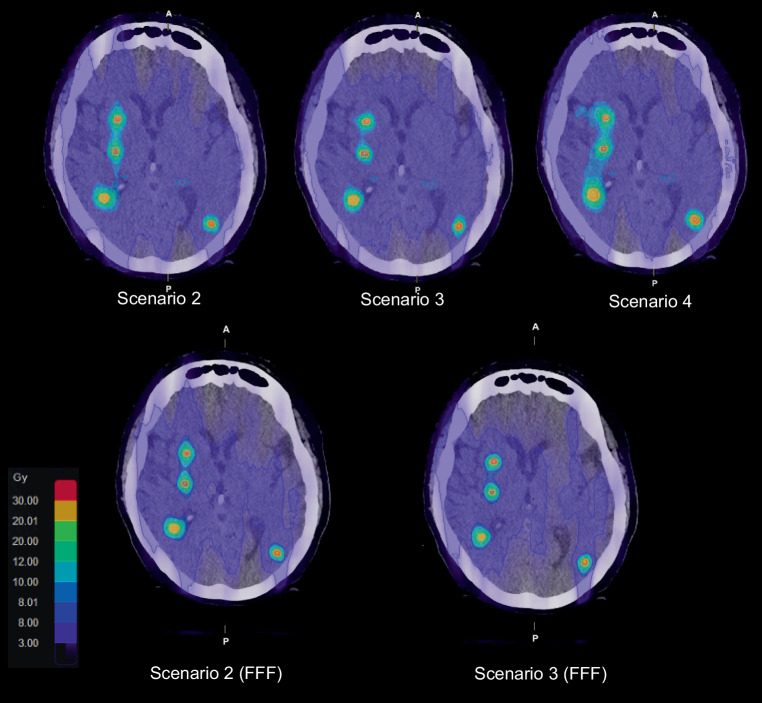
Dose distribution of a patient with 17 lesions for plans according to the scenarios 2, 3 and 4 with an energy of 6 MV (*upper row*) and scenarios 2 and 3 for 6 MV flattening filter free beams (FFF; *lower row*). The effects of a larger margin in scenario 4 for more distant metastases can be seen for the lesion in the left hemisphere

In order to get a better idea of the low dose distribution, we then evaluated the volume that had received 10% of the prescribed dose (V_10%_). Addition of arcs did not significantly change the V_10%_, but increasing the DCA complexity to a maximum in scenario 3 (427.72 ± 437.95 cm^3^) resulted in a 4% higher V_10%_ compared to scenario 1 (453.39 ± 430.2 cm^3^, *p* = 0.0024). Scenario 4 reached a V_10%_ of 514.4 ± 466.82 cm^3^ which was more than 10% higher compared to scenario 2 (464.28 ± 440.88 cm^3^, *p* < 0.0001). An example of how much the different scenarios (2–4) affect the low dose distribution is presented in Fig. 5.

### Subgroups

In the following section, the target volumes within each subgroup were examined to evaluate differences according to the number of lesions within a single plan. Since the changes for scenario 4 are consistent for all groups, arising from the larger PTV sizes and not from a change in planning settings, we just consider scenarios 1–3 in this comparison.

#### Few metastases (3–5)

For 3–5 metastases, a median of 8 arcs was used in scenarios 1 and 3, and significantly more (*p* = 0.008) in scenario 2, where in median 12 arcs were used. This addition did not significantly improve the quality of the plans in terms of monitor units, dose coverage and overdose. We found a significant difference in D_99%_ for scenario 1 vs. 3 (*p* < 0.017), but this difference would not be clinically relevant as it only amounts to an absolute difference of less than ±0.1 Gy for the mean values.

Local Paddick CI and GI were not improved by additional arcs only (scenario 1 vs. 2). Comparison of scenarios 1 and 3 showed slight improvement in CI (from 0.73 ± 0.08 to 0.74 ± 0.09 in scenario 3, *p* = 0.05); on the other hand GI increased (from 4.67 ± 1.23 to 4.88 ± 1.18 in scenario 3, *p* = 0.019).

Of all subgroups, we saw the least isodose overlaps in this group. This is associated with the lower number of lesions, since the number of missing GIs rises with the number of metastases (Fig. 4).

This subgroup did not differ in V_10%_ for scenarios 1, 2 and 3. Mean values were 142.15 ± 119.78 cm^3^, 141.22 ± 134.34 cm^3^ and 147.15 ± 132.35 cm^3^, respectively. Likewise, for global V_12Gy_ there were no significant differences between scenarios 1 vs. 2 and 1 vs. 3.

#### Medium number of metastases (6–10)

Only subgroup with 6–10 metastases showed a significant difference in MU for the comparison of scenario 1 with 2. Here, a significant increase from an average of 9597.83 MU to 12,546.83 could be observed, which however did not influence the dosimetric plan quality.

Neither of the modifications in the different scenarios improved global V_12Gy_.

The cumulated volume of the normal tissue exposed to low dose irradiation (global V_10%_) increased from 352.10 ± 83.73 cm^3^ in scenario 1 to 383.57 ± 72.51 cm^3^ in scenario 3 when the DCA complexity was limited (*p* = 0.036), while addition of arcs did not change the V_10%_. Increased PTV margins again lead to a greater volume of 431.14 ± 132.15 cm^3^ compared to scenario 2 (369.24 ± 88.78 cm^3^, *p* = 0.036).

For plans with 6–10 metastases, on average half of the lesions shared the 50% isodose with one or more other lesions, regardless of which scenario was chosen. As shown in Fig. 4, there was no difference in the number of missing GIs.

#### Many metastases (11–28)

Looking at the different scenarios for this subgroup with 11 or more metastases, a minimum of 10 and a maximum of 14 arcs was necessary for optimization of each plan.

We found no significant difference in D_99%_ between scenarios 1 and 2. Allowing additional arcs did not improve coverage or increase the number of arcs, as the maximum number of arcs was already exhausted. Also, no significant differences regarding monitor units were observed. As the number of lesions is very high, restriction of DCA complexity does not seem to have an impact on the number of monitor units in these cases. A statistically significant difference in D_99%_ was found between scenarios 1 and 3 (20.43 ± 0.37 Gy for scenario 1 vs. 20.34 ± 0.40 Gy for scenario 3, *p* = 0.032); however, this again would not be deemed clinically relevant.

Considering quality indices, the local GI was found to be significantly higher for scenario 3 (7.66 ± 1.71) compared with scenario 1 (7.36 ± 1.17, *p* < 0.005).

The V_10%_ was similar for all scenarios, with an average value between 1135.15 ± 165.15 cm^3^ for scenario 1 and 1165.73 ± 136.75 cm^3^ for scenario 3. On average more than half of the lesions shared the 50% isodose with one or more other metastases, regardless of the scenario and without statistically significant differences.

### Comparison with a flattening filter free beam

After replanning, we compared scenario 2 and 3 regarding the difference arising from applying a flattening filter free beam of the same photon energy.

With 6X FFF, global V_12Gy_ decreased significantly compared with the according 6X plans. On average scenario 2 yielded an 8.1% lower V_10Gy_ (25.39 cm^3^ for 6X and 23.33 cm^3^ for 6X FFF, *p* < 0.001) and a 7.5% lower V_12Gy_ (28.74 cm^3^ for 6X and 26.58 cm^3^ for 6X FFF, *p* = 0.0014) for FFF vs. flat beams. Yet no difference was apparent in the V_10%_ data.

There was no significant difference in local GIs between the 6X and 6X FFF plans, regardless of which scenario was used. Scenario 3 did not produce significantly different local Paddick CIs for the two energies, but for scenario 2 the difference was significant (*p* < 0.001) with a mean CI of 0.61 for 6X and a slightly lower mean Paddick CI of 0.60 for 6X FFF (statistically significant, but not clinically relevant).

For both scenarios, the number of monitor units increased for the FFF beam. While for a flattened beam on average 12,159.85 ± 6261.43 MU for scenario 2 and 12,265.40 ± 7328.33 MU were necessary, for an FFF beam the number rises by 14.7% for scenario 2 (13,951.15 ± 7573.42 MU, *p* < 0.001) and 13.1% for scenario 3 (13,866.45 ± 8612.68 MU, *p* < 0.001). The maximum number of monitor units was reached for scenario 3 and 6X FFF with 38,263 MU (patient #17, 28 metastases).

**Fig. 6:**
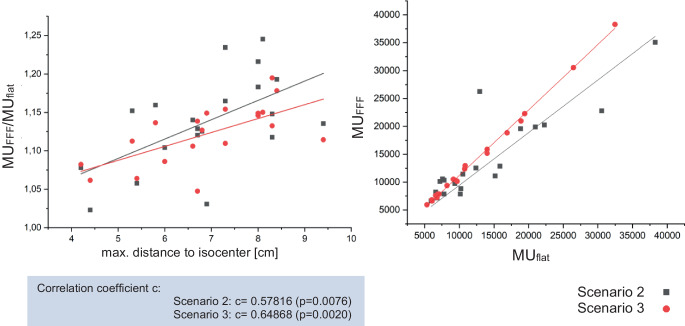
Number of monitor units needed for 6X FFF compared with 6X and number of monitor units needed for 6X FFF divided by the number of monitor units for 6X in dependence of the distance from the center of the lesion to the isocenter

The number of monitor units needed for 6X FFF compared with 6X increases in dependence of the maximum distance from the center of the lesion to the isocenter, i.e., the field size. This is shown in Fig. 6.

To determine to what extent the increased amount of monitor units for the FFF plans is counteracted by the higher dose rate, we measured the actual treatment time on our linac for a representative subset of plans. The flattening filter free plans required approximately half of the total delivery time for treatment compared to the regular 6X plans, for example 13.95 min instead of 26.55 min and 16.85 min instead of 31.85 min (patient #16 with 6 metastases and patient #7 with 17 metastases, respectively).

## Discussion

Our study showed that the different scenarios resulted in only minor differences in plan quality. This holds true for the analysis of all patients regardless of the number of metastases, where few significant differences were observed. It is crucial to highlight that these are highly intricate cases and we only evaluate the dose distribution without judging the clinical acceptability of the plans. Our focus was on the individual planning parameters and differences for the different planning strategies for selected scenarios with some parameters always fixed and not all optimizer possibilities used.

When considering the individual subgroups, we saw more significant differences, in particular how the increasing number of metastases affects the individual parameters. As expected, we found that with increasing number of lesions within a plan, the number of monitor units increased, so did the dose to healthy tissue and, in particular, the number of dose bridges between metastases. This is sensible, considering the larger irradiated cumulative volume. Accordingly, local CI and GI also deteriorate for more lesions.

Within the subgroups, the differences in parameters were most noticeable when comparing scenario 2 and scenario 4, as the larger margins already resulted in significantly larger cumulative target volumes and thus significantly increased the V_12Gy_ and V_10%_, i.e., the dose to the healthy brain tissue, as well as the dose to the hippocampus [14], which is intrinsic since a larger cumulative volume needs to be irradiated.

However, larger target volumes are easier for the algorithm to optimize, especially due to the limitations of the linac, and therefore better local Paddick CIs were achieved for this scenario. The same is true for the local GI, as a better gradient could also be achieved due to the larger target volumes [14]. In practice, since the target volumes were different for scenario 4, the validity of comparing local CI and GI can be called into question since this does not reflect true “plan quality”. It should also be noted that increasing the margins to achieve better plan metrics should never be the aim for SRS treatments, but rather reducing margins where possible. We have included the different margin rule because it may be an approach chosen by institutions concerned about positioning uncertainties of targets farther from the isocenter [23, 24]. However, increasing the margins will imply increased dose to healthy tissue. Therefore, the aim of SRS treatments must be precise localization and better intrafractional motion management, e.g., by using ExacTrac (Brainlab, Munich, Germany), 6‑degrees of freedom couch, surface monitoring, end-to-end tests and as a result less irradiation of healthy tissue.

Scenario 2 resulted in lower global V_12Gy_, V_10%_ and hippocampus dose compared to scenario 3, representing the case with least degrees of freedom. Even tough CI was slightly worse for scenario 2 (0.61 ± 0.12) in relation to scenario 3 (0.62 ± 0.13), a better GI was obtained with 5.97 ± 1.69 and 6.15 ± 1.71, respectively. The number of overlapping isodoses was also lower for the more complex plan, while the number of monitor units did not change significantly. However, it should always be evaluated whether these complex plans are deliverable, since at the point where limits of the linac are reached, for example the limit of the MLC speed, reality can differ from the plan. As the differences in plan quality are so small, less complex plans could in some cases be of advantage (see scenario 1 vs. 2).

**Fig. 7:**
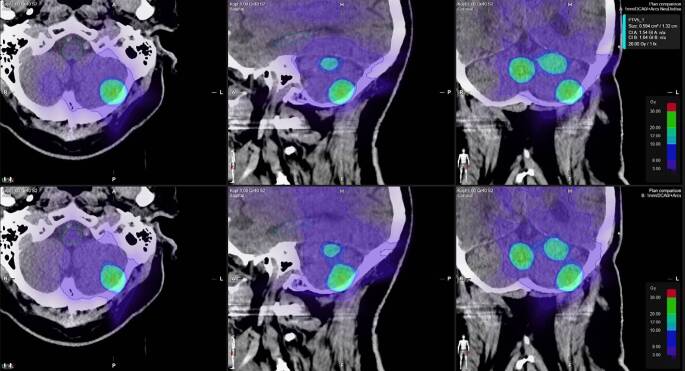
The *upper row* shows the dose distributions of a plan where extra arcs were added to the lesions farthest from isocenter, the *lower row* shows scenario 2 with extra arcs for the largest lesions

Addition of arcs for the three largest lesions (scenario 2) achieved no significant differences compared to the basic plan (scenario 1), so it should individually be evaluated whether additional arcs are really required. In practice, a first optimization without additional arcs should be performed. Then arcs should be added to those lesions that show insufficient metrics or dose bridges. This was not possible in this study since a fixed and reproducible scheme was used rather than individual optimization. It may be argued whether extra arcs should be added not for the largest metastases, but rather for those most distant from the isocenter. To check the effect of this approach, we recalculated a number of example cases with this modified strategy. The difference in plan metrics as well as dose distributions was relatively minor (Fig. 7), so that a detailed comparison was not presented in this manuscript. Our choice to add the arcs to the largest metastases was meant to compensate for the fact that the Elements version used in this work does not allow for VMAT optimization, which we expect to be most beneficial for larger target volumes. However, in the clinical practice there may be cases (particularly with FFF beams) in which more distant metastases or lesions located close to organs at risk may benefit most from extra arc. This should be considered when optimizing the individualized plan, but falls outside the scope of the “planning recipe approach” of the present study.

The greatest differences between all scenarios were found in the dose bridges, i.e., the distribution of the 50% isodose in the individual plans. These differences were readily apparent to the eye on visual inspection of the plans, but were hardly reflected in the numerical metrics of plan quality which were statistically evaluated.

Changing the energy from a standard flattened beam to an unflat 6X beam resulted in significantly lower global V_12Gy_ for both scenarios, but no clinically relevant changes in V_10%_, local CI or GI were achieved. Even though the number of monitor units went up by more than 13% [19], the higher dose rate compensates for this shortfall, logically resulting in drastically shorter treatment times (on average −46%). The further the maximum distance from the center of a lesion was from the isocenter, the more MU were needed for 6X FFF plans compared to 6X. This is conclusive, arising from the nature of a flattening filter free beam, where the peripheral dose is reduced relative to the central axis.

**Fig. 8:**
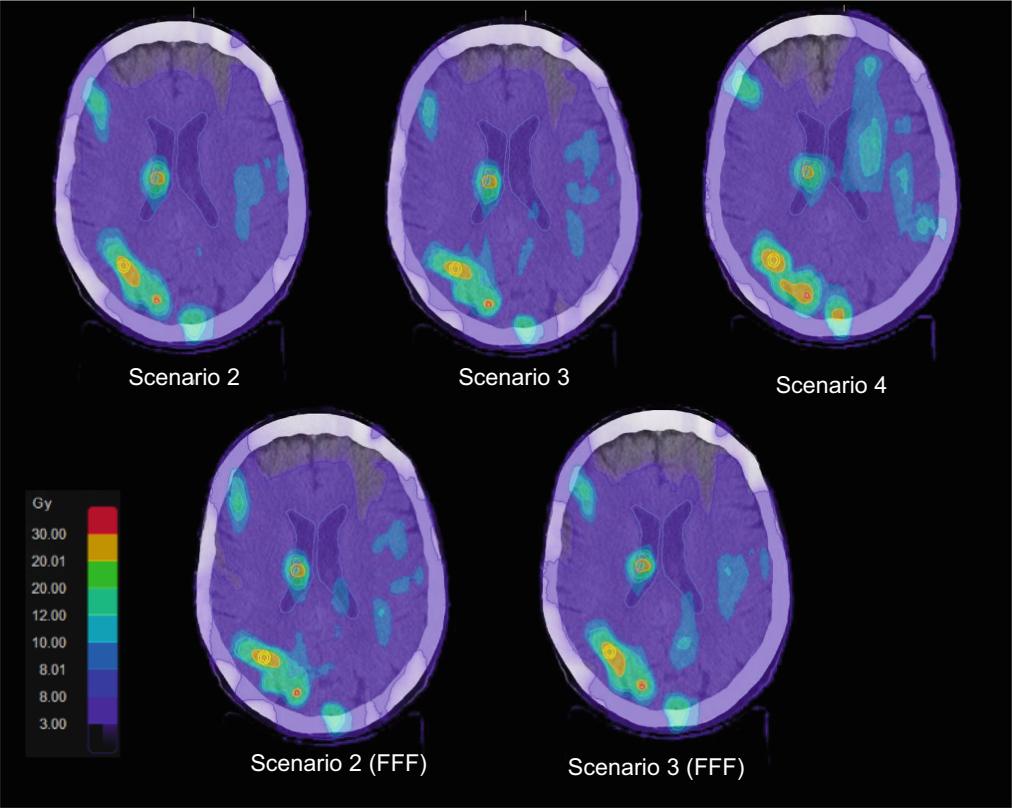
Dose distribution of a patient with 28 lesions for plans according to the scenarios 2, 3 and 4 with an energy of 6 MV (*upper row*) and scenarios 2 and 3 for 6 MV flattening filter free beams (FFF; *lower row*)

Even though no clinically relevant differences in parameters such as global V_12Gy_, local CI and GI where found, we identified some variation in the dose distributions. These differences appeared between the dose distributions of the different scenarios, while for 6X and 6X FFF of the same scenario the distributions were similar. We measured the width of several overlapping 50% isodoses for scenarios 2 and 3 for both energies and did not find a correlation between the widths, but saw that certain scenarios generated less overlapping 50% isodoses. For example, for patient #15 with 17 metastases (Fig. 3), we saw that for scenario 2 and both energies five metastases shared the 50% isodose, while for scenario 3 with 6X and the same metastases only four and for the 6X FFF version only three metastases clustered and shared a V_10Gy_. The different dose distributions can also be clearly seen for patient #17 with 28 metastases in Fig. 8.

Therefore, from a clinical point of view a more complex scenario may in the end be preferred although the differences in the quality metrics appear minor. Judging only from the absolute value of the metrics or a ‘green/yellow/red light approach’, scenario 1 seems to be a good choice if fast and straightforward plan optimization and short treatment times are needed. Parameters such as global V_12Gy_, V_10%_ indicate good plan quality. However, this scenario generated the most dose bridges and visually less optimal isodose distribution. In some cases, this might affect the decision to accept this plan for treatment.

For other planning options such as HyperArc in Eclipse, which uses a VMAT technique instead of dynamic conformal arcs, different plan quality metrics might be achieved. This question is subject for further research and is especially interesting in comparison with Elements MBM.

It is worth mentioning that this study considered the optimization scenarios in a standardized fashion without further individual optimization and iteration as the aim of this study was to provide a reproducible set of plans for a consistent comparison of the most relevant parameter settings. Consequently, good comparability of the plans was a prerequisite. In the clinical setting, these plans would be a starting point, then the planner might decide to proceed to further iteration steps where needed. Furthermore, a new version (MBM 4.0) has recently been released, but despite updated algorithms, we believe that many of the conclusions are transferrable to newer versions, especially considering the speed with which they are released.
